# Interval between contrast administration and T1-weighted MRI for cerebral adrenoleukodystrophy: a single-case observation

**DOI:** 10.1186/s41747-023-00373-6

**Published:** 2023-10-02

**Authors:** Marco Moscatelli, Chiara Benzoni, Fabio M. Doniselli, Mattia Verri, Riccardo Pascuzzo, Domenico Aquino, Federica Mazzi, Alessandra Erbetta, Ettore Salsano

**Affiliations:** 1 Neuroradiology Unit, Fondazione IRCCS Istituto Neurologico Carlo Besta, Via Celoria 11, 20133 Milan, Italy; 2 Department of Biomedical Sciences for Health, University of Milan, Via Mangiagalli 31, 20122 Milan, Italy; 3 Unit of Rare Neurological Diseases, Fondazione IRCCS Istituto Neurologico Carlo Besta, via Celoria 11, 20133 Milan, Italy

**Keywords:** Adrenoleukodystrophy, Contrast media, Gadobutrol, Gadolinium, Magnetic resonance imaging

## Abstract

In adrenoleukodystrophy (ALD), contrast enhancement (CE) is a disease activity marker, but there is uncertainty about the optimal delay, if any, between contrast injection and magnetic resonance imaging (MRI) acquisition to avoid false-negative results. We acquired axial two-dimensional (2D) and three-dimensional (3D) T1-weighted gradient-echo every 6 min from 0 to 36 min after contrast administration (gadobutrol 0.1 mmol/kg) in an ALD patient with enlarging white matter lesions and progressive neuropsychological symptoms, using a 3-T magnet. The image signal over time was qualitatively assessed and measured in two regions of interest. On 3D sequences, no definite CE was appreciated, whereas on 2D sequences, CE was noticed after 6 min and definitely evident after 12 min, when 73% of the maximum signal intensity was measured. In ALD subjects, contrast-enhanced 2D T1-weighted gradient-echo sequences acquired at least 10 min after contrast injection may be considered to reduce false negative results.

**Relevance statement**

Our report is the first attempt to find an optimal delay between contrast administration and T1-weighted acquisition in cALD patients in order to correctly detect disease activity and avoid false negative results.

**Key points**

• The optimal time between contrast injection and image acquisition for MRI of adrenoleukodystrophy is unknown.

• Contrast enhancement predicts adrenoleukodystrophy progression and could help patient’s selection for the therapy.

• We acquired two post-contrast T1-GRE-2D/3D sequences several times to find the best injection-time.

• T1-weighted 2D GRE resulted more sensitive than T1-weighted 3D GRE even after long intervals from injection.

• A delay of about 10 min may minimize false negatives.

**Graphical Abstract:**

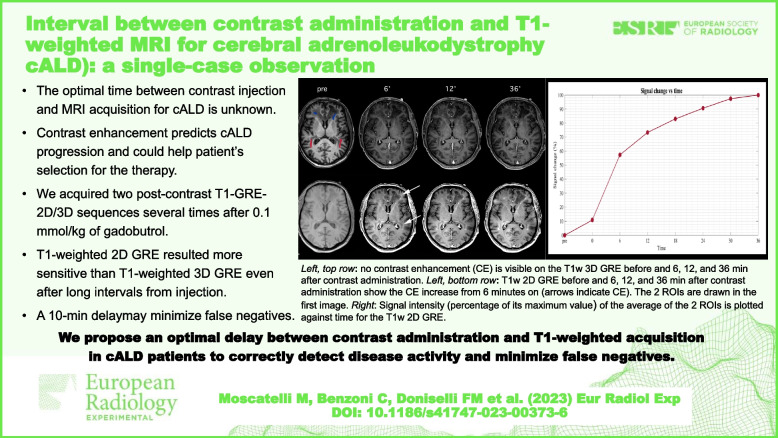

## Background

Adrenoleukodystrophy (ALD) is an X-linked peroxisomal disease caused by mutations in the *ABCD1* gene, which lead to the accumulation of very long chain fatty acids. In males, ALD encompasses a variety of clinical phenotypes, including cerebral ALD (cALD) and pure adrenomyeloneuropathy (pAMN). The most aggressive cALD is typically seen in children, and, at onset, it is characterized by neuropsychological symptoms combined with cerebral demyelination on magnetic resonance imaging (MRI). The milder pAMN is typically seen in adults, and, at onset, it is characterized by neurological symptoms (*i.e.,* spastic-ataxic gait and bladder dysfunctions) combined with normal brain MRI. Patients with pAMN can progress to cerebral AMN (cAMN), *i.e.,* they can develop cerebral demyelination and survive as poorly as the children with cALD [1, 2].

In the presence of cerebral WM abnormalities suggestive of demyelination, contrast-enhanced T1 sequences are essential for the management of the disease [3, 4]. Contrast enhancement strongly predicts disease progression, reflecting inflammation associated with blood-brain barrier breakdown, and its mere presence has been deemed necessary for a patient with cerebral demyelinating lesions to be candidate for hematopoietic stem-cell gene therapy or hematopoietic stem-cell transplantation [5, 6], the only disease-modifying therapies for cALD/cAMN available so far [1]. However, the optimal timing and sequences for post-contrast T1 imaging are still a matter of debate [4, 7].

Here, we investigated a patient with adult-onset cALD in order to determine an optimal post-contrast T1 imaging for the detection of contrast enhancement in this disease.

## Methods

**Fig. 1:**
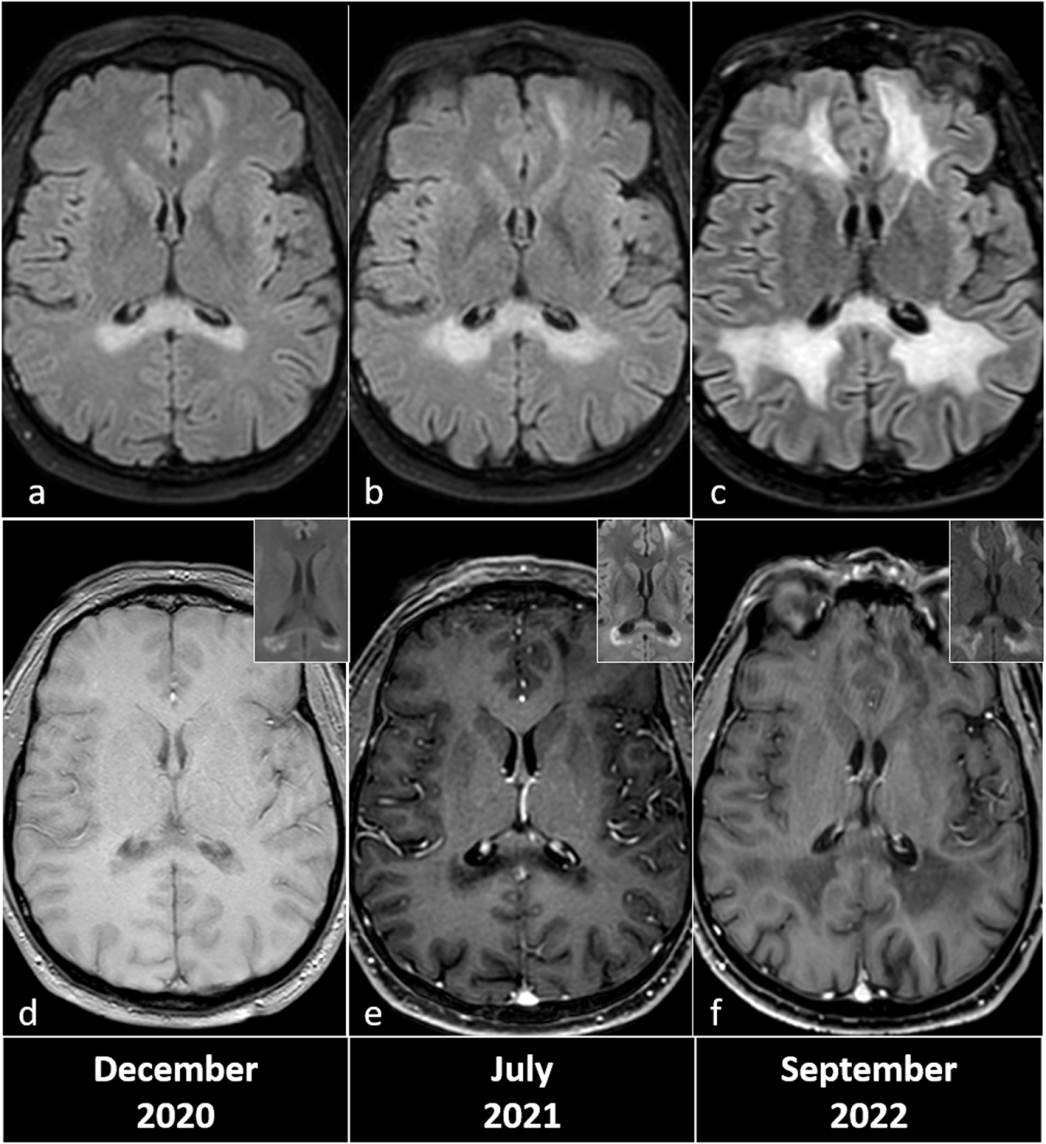
Progression of cerebral white matter abnormalities in a 38-year-old patient affected with adrenoleukodystrophy. Axial fluid-attenuated inversion recovery (FLAIR) images show a slight increase in lesions from September 2020 (**a**) to July 2021 (**b**) and an abrupt progression in September 2022 (**c**). A three-dimensional T1-weighted gradient-echo sequence failed to demonstrate enhancement in July 2021 (**e**) and September 2022 (**f**), while on September 2020, magnetic resonance imaging was performed without contrast injection (**d**, axial T1-weighted image). High *b*-value on diffusion-weighted imaging constantly shows a fringe of potential disease activity at the edge of lesions (insets in **d**, **e**, and **f**)

On August 2017, a 38-year-old asymptomatic man underwent *ABCD1* genetic testing, since he had a family history of ALD. The analysis revealed that he was carrier of the familial *ABCD1* pathogenic variant (c.838C>T; p.Arg280Cys) [8]. On December 2020, he agreed to undergo his first brain noncontrast MRI, which showed WM abnormalities suggesting progressive cALD (Fig. 1a–d). He was still asymptomatic, but he refused to consider hematopoietic stem-cell transplantation. On July 2021, routine brain MRI including contrast-enhanced sequences displayed a slight progression of the WM abnormalities but no detectable enhancement (Fig. 1b–e). On May 2022, he had a minor head trauma caused by car accident. One week later, he started to have his first symptoms consisting of progressive cognitive and behavioral abnormalities. On September 2022, brain MRI including contrast-enhanced sequences showed marked WM abnormalities progression, still without visible enhancement (Fig. 1c–f). Strikingly, we noticed that the edge of these expanding WM abnormalities was characterized by a rim of diffusion restriction (Fig. 1, insets).

On October 2022, the patient was admitted to our Institute for further evaluation. Given the marked WM abnormalities progression, we deemed that the absence of enhancement could be due to a short interval between contrast injection and MRI scan. Hence, we decided to scan the patient also at longer than usual time-points after contrast administration. Specifically, on a 3-T scanner (Achieva, Philips Healthcare, Best, the Netherlands), we acquired a set of two sequences with a 32 channels head-coil: an axial T1-weighted two-dimensional (2D) gradient-echo (GRE) sequence (fast field-echo, echo time 4.6 s, repetition time 349.4 s, number of excitations 2, flip angle 80°, acquisition time 2.02 min, filed of view 230 × 200 mm, pixel size 0.68 × 0.85 mm, matrix 340 × 235, slice thickness 4 mm, reconstructed pixel size 0.45 × 0.45 mm, 32 slices, gap 0.3 mm, bandwidth 345 Hz) and a T1-weighted three-dimensional (3D) GRE sequence (fast field-echo, echo time 3.9 s, repetition time 8.4 s, number of excitations 1, flip angle 8°, acquisition time 4.07 min, filed of view 256 × 256 mm, matrix 268 × 268, pixel size 0.96 × 0.96 mm, slice thickness 1.15 mm, reconstructed pixel size 1.05 mm, 306 slices, gap -0.37 mm, bandwidth 740 Hz), in this order. We repeated this set before contrast administration (manual injection of 0.1 mmol/kg of gadobutrol, Gadovist® 1.0 mmol/mL, Bayer AG, Leverkusen, Germany) with a subsequent saline flush of the same volume), immediately after, and after an interval of 6, 12, 18, 24, 30, and 36 min.

Apart from subjective image evaluation, we drew two regions of interest (ROIs) at the edge of the lesions (where blood-brain barrier breakdown is usually caused by inflammation) in frontal and parietal lobes, using ITK-SNAP software (http://www.itksnap.org/). Then, we plotted the average signal intensity of the two ROIs against time for 3D and 2D images.

As this was a single-subject case study, no statistical inference analyses were necessary.

## Results

**Fig. 2:**
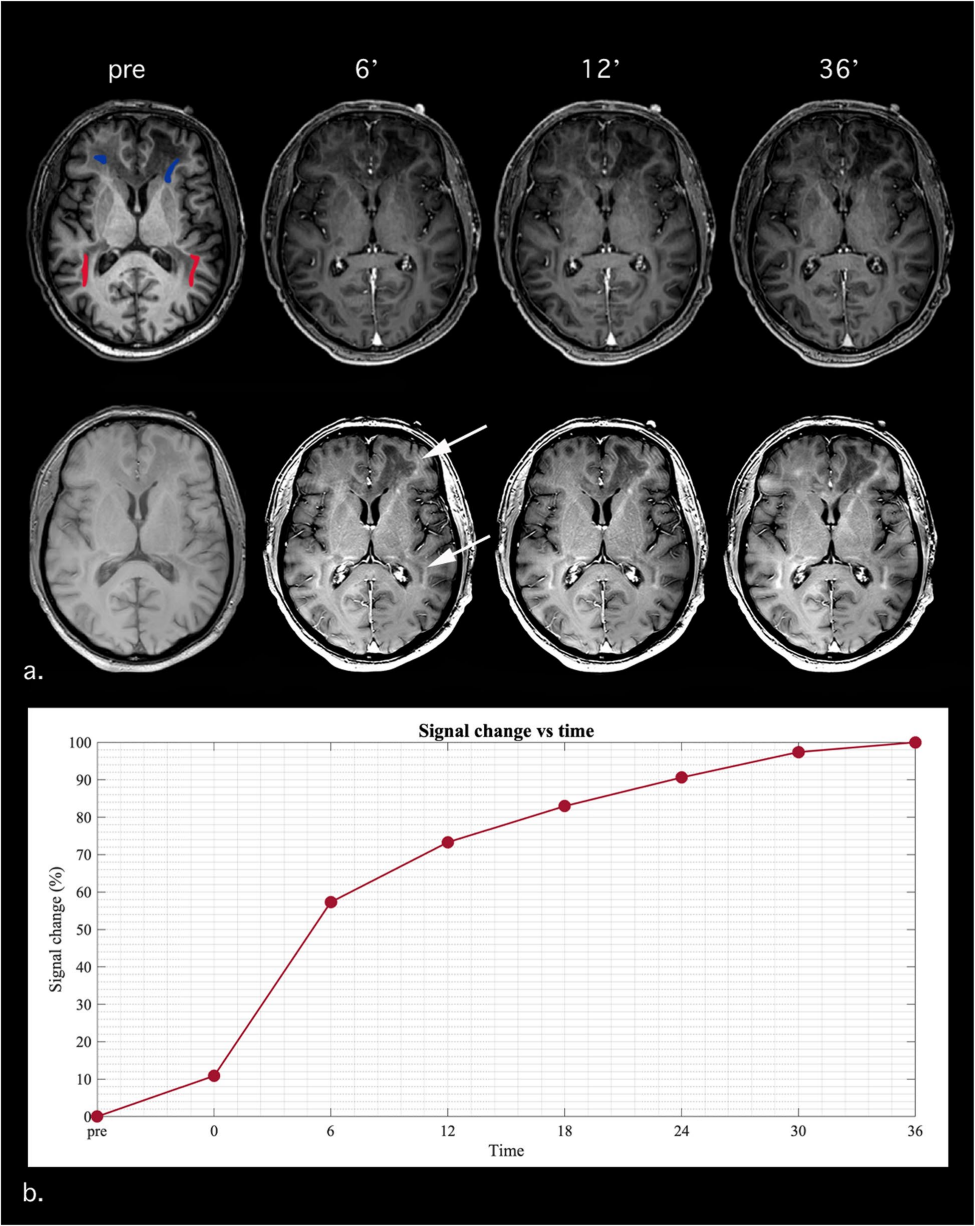
Variation of contrast enhancement detection on different T1-weighted sequences at different time points in the same adrenoleukodystrophy patient shown in Fig. 1. **a, top row:** no contrast enhancement is visible on the T1-weighted three-dimensional (3D) gradient-echo sequence before and 6, 12, and 36 min after gadobutrol administration. **a, bottom row:** the T1-weighted two-dimensional (2D) gradient-echo sequence before and 6, 12, and 36 min after gadobutrol administration show the presence of contrast enhancement from 6 minutes on; the arrows indicate the areas of enhancement on the 6-min image. **b** Signal intensity variation, expressed as percentage of its maximum value, is plotted against time for the T1-weighted 2D gradient-echo sequence as average of the two regions of interest (ROIs) drawn on the precontrast image of the 3D series (one ROI in the frontal lobes, considering two blue areas; one ROI in the parietal lobes, considering two red areas) to allow a clean evaluation of the 2D series

On T1-weighted 3D GRE sequences, no definite enhancement was seen at any time point (Fig. 2a, top row), whereas on T1-weighted 2D GRE sequences, the enhancement was detectable at 6 min after injection, became definitely visible at 12 min, and reached the maximum at 36 min (Fig. 2a, bottom row). Accordingly, the signal intensity analysis showed that the curve slope remained quite flat on T1-weighted 3D GRE sequences (not shown), whereas on T1-W 2D GRE images, the enhancement reached 56% of its maximum 6 min after the contrast injection, 73% of its maximum after 12 min, and the plateau after 36 min (Fig. 2b).

## Discussion

Contrast-enhanced T1-weighted MRI is fundamental for common neurological diseases that can be characterized by blood-brain barrier breakdown, such as multiple sclerosis (MS) [9] and brain tumors [10]. In those diseases, the presence, or absence, of gadolinium enhancement can have important diagnostic, prognostic, and therapeutic implications, while the possibility of false-negative MRI scans after contrast injection represents a main issue. In ALD, this issue has been poorly addressed, and there is uncertainty about the proper T1-weighted sequence and delay of acquisition from contrast injection [4, 7].

T1-weighted 2D turbo spin-echo and T1-weighted 3D magnetization-prepared rapid acquisition by gradient echo, called MPRAGE, have been recently demonstrated to be both valuable in allowing to visualize contrast enhancement in pediatric cALD, although T1-weighted 2D turbo spin-echo seems to be more sensitive for low degree of enhancement [7].

In this experience regarding a single patient with progressive cALD, the detection of contrast enhancement seemed to be dependent on the type of T1-weighted sequences, with the 2D GRE sequence appearing more sensitive than the 3D GRE sequence even after long intervals from injection. We also showed that contrast enhancement, when present, may be definitely visible from 12 min on. The greater slice thickness in the axial 2D sequence could provide higher signal-to-noise ratio (SNR), thus increasing image quality and making contrast-medium enhancement more conspicuous. For this reason, 2D sequences could detect subtle changes associated with contrast leakage compared to the 3D sequences.

The ancillary observation of restriction at the edge of the active lesions in diffusion-weighted images could represent the front of active demyelination led by a fringe of inflammatory cells [11]. This phenomenon has been previously mentioned [12] but not fully addressed and could become a biomarker of disease activity.

Only one patient was examined given the rarity of the condition, and spin-echo sequences were not included in the protocol because of time constraints. Further, it is worth noting that in cases of progressive cALD, contrast enhancement is usually seen also on T1-W 3D GRE sequences and at a shorter delay, as shown by Cebeci et al. [7], and also according to personal observations of the authors of the current report. Despite these considerations, however, our experience, even though based on a single-patient observation, suggests that in ALD subjects, contrast-enhanced T1-weighted 2D GRE sequences acquired at least 10 min after the contrast injection should be considered to minimize the risk of false-negative results.

## Data Availability

The data presented in this study are available on request from the corresponding author.

## Abbreviations

2D: Two-dimensional
3D: Three-dimensional
ALD: Adrenoleukodystrophy
cALD: Cerebral ALD
cAMN: Cerebral AMN
CE: Contrast enhancement
GRE: Gradient-echo
MRI: Magnetic resonance imaging
pAMN: Pure adrenomyeloneuropathy
WM: White matter
